# Quality and Phytochemical Composition of Sweet Cherry Cultivars Can Be Influenced by Altitude

**DOI:** 10.3390/plants12122254

**Published:** 2023-06-09

**Authors:** Diana Nacouzi, Rim Masry, Walid El Kayal

**Affiliations:** Faculty of Agricultural and Food Sciences, American University of Beirut, P.O. Box 11-0236, Beirut 1107, Lebanon

**Keywords:** sweet cherries, altitude, maturity indices, phytochemicals

## Abstract

Sweet cherries (*Prunus avium* L.) are among the most important stone fruits in Lebanon. They are harvested between May and July; however, the introduction of new early varieties in low and medium altitudes (500–1000 m) and late varieties in higher altitudes (1800–2200 m) along with postharvest technologies can extend harvesting season. In this study, physicochemical characteristics along with total phenolic content, total anthocyanin content, and antioxidant activity of the most commercial cherry cultivars were evaluated at different altitudes to determine optimum harvesting time. Findings indicated that some varieties, such as “Teliani” and “Irani”, are more significantly impacted by altitude than the other varieties in terms of maturity indices. Duration of fruit development was prolonged with altitude, and in most instances, higher fresh weights and sizes were observed; however, fruit firmness decreased. While total phenolic content (expressed as gallic acid equivalent) did not significantly vary between varieties, the antioxidant activity (FRAP and DPPH assays) showed the lowest value in “Banni” and the total anthocyanin content showed the highest levels in “Irani” and ”Feraouni” and the lowest in “Mkahal” and “Banni”. Furthermore, total phenolic content and reduction of ferric complex (FRAP) were interestingly influenced by geographical locations, in contrast to total anthocyanin content and radical scavenging activity (DPPH) which were unaffected.

## 1. Introduction

Sweet cherry (*Prunus avium* L.) is one of the most important non-climacteric fruit crops of the Rosaceae family. It is produced worldwide in temperate climate regions [1] with expanding agronomic and economical status [2]. In Lebanon, sweet cherry production is increasing highly linked by the Mediterranean climate that allows farmers to achieve a profitable sweet cherry production [3]. According to recent statistics, the Lebanese cherry production is about 33,683.86 tons per year planted over a surface of 4294 Ha (FAOSTAT, 2021) [4] in many regions of the country, mainly in the Bekaa valley and Mount Lebanon. Sweet cherry production has been subjected to a great increase in the last decade with recent introduction from abroad of potential new cherry cultivars and rootstocks with unknown properties. By using different cultivars, farmers can extend the harvest window and avoid lower returns during the peak harvesting period. Cherries are perishable non-climacteric fruits; thus, the determination of the optimum harvesting time is the most important factor for storage and marketing purposes especially if farmers are targeting global markets. Additionally, the perspective of a quality-oriented crop evaluation is mainly to favor access to international markets that have well defined export standards.

Sweet cherries display noteworthy diversity in various traits such as fruit size, shape, color, sugar content, flowering time, and defense mechanisms against pathogens. These agronomic traits are responsible for the crop’s adaptation, which led to the development of genetic variability in sweet cherries. Many studies have characterized their genetic diversity and identified genes controlling traits of interest, which was considered a key factor for sweet cherry breeding programs [2]. 

In the last few years, the physicochemical characteristics of sweet cherry varieties were studied around the world. These characteristics define the fruit quality and determine correlations between fruit properties. Adequate evaluation of Lebanese sweet cherry cultivars has yet to be carried out. Since cherries are cultivated on different altitudes in Lebanon, ranging from 900 m to 2000 m above sea level, climatic and geographical influences can be considered as important factors affecting fruit quality and thus having a remarkable impact on marketing. Many attributes such as fruit size, skin color, flavor, sweetness, sourness, firmness, and fruit color determine the quality of sweet cherries in the market, and they are highly related to consumers’ acceptability [5]. As sweet cherry is a non-climacteric fruit, harvested at the stage of consumption maturity, with relatively high levels of respiration rate [6], harvesting time is very crucial for storage and marketing purposes.

However, cherries are considered perishable fruits with short shelf life; therefore, during their cold storage, severe losses can occur caused by fruit transpiration, physiological disorders, and fungal diseases that may disqualify the fruits during sorting and packaging [7]. In addition, agricultural practices adopted by farmers may have different consequences on postharvest fruit quality and hence, its storage [8]. Nonetheless, sweet cherry fruit properties can be advantageous for breeding programs, crop production, and storage technologies [9].

Sweet cherries are highly susceptible to fruit cracking usually induced by rain. Consequently, in specific epidermal regions, water uptake is concentrated by microcracks, resulting in bursting of the fruit flesh cells and leakage of cell contents into the apoplast [10]. Some studies proved that cherry fruit cracking and rots were reduced significantly by using rain cover, depending on cultivar and season in which saleable fruits varied from 56.9 to 99.0% under rain cover. Cherry fruits under rain covers could be harvested in the best ripening period as well as in rainy season [11].

Another significant physiological issue in sweet cherry is non-uniform fruit maturity, which can be attributed to a low flower bud initiation level regulated by hormone signals and several enzymes. As a result, there will be a decrease in changes from the vegetative to the reproductive state of the cherry tissue. Consequently, this delay may be significant increases in age differences, flowering, and cherry fruit maturation [3]. The ideal time for initiation of fruit quality properties is defined by the variations in skin color and the increase of glucose and fructose contents with an important increase in fruit size [3]. Sugars have a major role in quality of sweet cherry fruits since they are balanced with acids in the fruit to give flavor. Levels of sugars can reach 25 g/100 g of fruit. The most important types of sugars that are usually present in sweet cherries are glucose and fructose mainly (90% of total sugars of the fruits), sucrose, maltose, and sorbitol and their levels vary according to the cultivar, agronomic factors, environmental conditions, and the stage of development and ripening [12]. According to Crisosto et al. [13], a strong relationship exists between customers’ acceptability and the levels of sugars in the fresh cherry fruit.

Together with the significance of their physiochemical characteristics, sweet cherries have several phytochemicals that add to their nutritional value and health benefits. Anthocyanins, which have been linked to a range of health benefits including anti-inflammatory, anti-cancer, and cardiovascular protection, are abundant in sweet cherries. According to research by Simunic et al. [14], sweet cherries contain 17 distinct anthocyanins, the most prevalent of which is cyanidin-3-glucosyl-rutinoside. Sweet cherries also contain a lot of flavonols, such quercetin, which has anti-inflammatory and antioxidant qualities. A study by Jang et al. [15] found that quercetin was the predominant flavonol in sweet cherries. Interestingly, the exact types and amounts of polyphenols in sweet cherries can vary depending on the variety and growing conditions. For example, González-Gómez et al. [16] analyzed the phenolic composition of six different sweet cherry cultivars grown in Spain. The results showed that the total phenolic content varied between the cultivars, with the highest levels found in the “Sweetheart” and “Lapins” varieties.

There is little information available on the changes in quality criteria that occur during the ripening phase of sweet cherries and their nutritional and antioxidant properties. It is consequently vital to have maturity indicators available that indicate the optimal harvesting period so that the fruit retains its physicochemical and sensory qualities.

The aim of this study is to assess the fruit quality of the main sweet cherry cultivars growing at different elevations in Lebanon for fresh consumption throughout the repining season by measuring their agro-morphological characteristics such as skin color, fruit firmness, dry matter, fruit diameter, weight, titratable acidity, total soluble solids, and initial pH. Moreover, total phenolic content, anthocyanins and antioxidant capacity were quantified to evaluate the biological activities of these varieties as an important step to enhance sweet cherries breeding programs that might lead to developing new promising cultivars with enhanced cytotoxic properties against potential human diseases.

## 2. Results

### 2.1. Physico-Chemical Analysis

**Figure 1:**
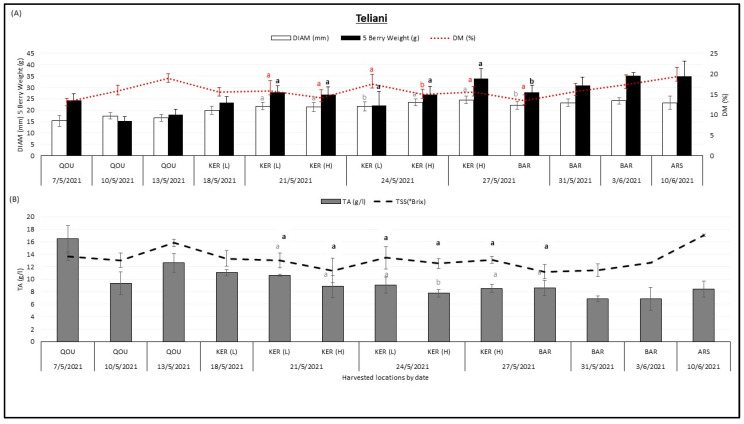
Variation of fruit diameter DIAM (mm), 5 berry weight (g), dry matter DM (%) (**A**), titratable acidity TA (g/L), and total soluble solids TSS (°Brix) (**B**), in “Teliani” cherry variety between different locations Qoussaya (QOU), Kaa El Reem high altitude (KER H), Kaa El Reem low altitude (KER L), Barqa (BAR), and Arsal (ARS) over the harvesting season. While comparing different locations at the same date, mean values followed by the same letter are not significantly different at *p* ≤ 0.05 level. Variables were considered significant <0.05 risk level.

Sweet cherries are a highly perishable fruit and their quality deteriorates rapidly after harvest. Therefore, it is important to determine the optimal time of harvest and the appropriate postharvest handling practices to maintain the quality and extend the shelf life of cherries. Data on physicochemical characteristics are presented per variety from all locations where this variety is planted during its ripening season. Figure 1 describes the variation of fruit diameter DIAM (mm), 5 berry weight (g), dry matter DM (%), titratable acidity TA (g/L), and total soluble solids TSS (°Brix) in “Teliani” cherry varieties between different locations over the harvesting season.

The “Teliani” variety was harvested from Qoussaya (QOU), Kaa El Reem high altitude (KER H), Kaa El Reem low altitude (KER L), Barqa (BAR), and Arsal (ARS) between 7 May 2021 and 10 June 2021. It was observed that the 5 berry weight (g) of “Teliani” variety tended to increase with the season. The lowest 5 berry weight (15.14 g) was found in Qousaya on 10 of May 2021 whereas the highest 5 berry weight (35 g) was found in Barqa on 3 June 2021. Fruit diameter (mm) tended to increase between May and June, the lowest fruit diameter values (15.27 mm) were recorded in Qousaya on 7 May 2021 while the highest fruit diameter values (24.49 mm) were recorded in Kaa El Reem (H) on 27 May 2021. Dry matter (%) and sugar content tended to increase with the season and their values almost followed a similar trend. The lowest dry matter (13.18%) was found in Qousaya on 7 May 2021 whereas the highest dry matter (19.26%) was found in Arsal on 10 June 2021. The lowest TSS (11.2 °Brix) was found in Barqa on 27 May 2021 whereas the highest TSS (17 °Brix) was found in Arsal on 10 June 2021. Only titratable acidity (g/L) tended to decrease with harvest date, the lowest TA (6.86 g/L) was found in Barqa on 3 June 2021 whereas the highest TA (16.5 g/L) was found in Qousaya on 7 May 2021. When comparing “Teliani” variety in the same harvest date between two different locations, significant differences were observed on 24 May 2021 for fruit diameter, dry matter, and TA between Kaa El Reem (L) and Kaa El Reem (H), and on 27 May 2021 for fruit diameter and berry weight between Kaa El Reem (H) and Barqa.

Table 1 illustrates the variation of fruit diameter DIAM (mm), 5 berry weight (g), dry matter DM (%), titratable acidity TA (g/L), and total soluble solids TSS (°Brix) in “Skeena” and “Banni” cherry varieties over the harvesting season. The “Skeena” variety was harvested only from Kaa El Reem high altitude, between 21 June and 2 July 2021. It was shown that the 5 berry weight (g) of the “Skeena” variety tended to slightly increase with the season. The lowest 5 berry weight (46 g) was found on 21 June 2021 whereas the highest 5 berry weight (54 g) was found on 24 June 2021. Fruit diameter (mm) tended to slightly increase between June and July, the lowest fruit diameter values (26.12 mm) were recorded on 24 June 2021 while the highest fruit diameter values (27.26 mm) were recorded on 2 July 2021. Dry matter (%) and sugar content tended to slightly increase with the season and their values almost followed a similar trend. The lowest dry matter (17%) was found on 21 June 2021 whereas the highest dry matter (20%) was found on 2 July 2021. The lowest TSS (16.5 °Brix) was found on 24 June 2021 whereas the highest TSS (20 °Brix) was found on 28 June 2021.Titratable acidity values (g/L) were more stable with harvest date, the lowest TA (9.27 g/L) was found on 24 June 2021 whereas the highest TA (9.67 g/L) was found on 28 June 2021.

The “Banni” variety was harvested from Kaa El Reem high altitude and Arsal between 21 June 2021 and 8 July 2021. The 5 berry weight (g) of “Banni” variety tended to increase with the season in the same location, but an important difference was observed between Kaa El Reem (H) and Arsal due to the fact that cherry orchards in Arsal are not irrigated but kept rainfed throughout the season. The lowest 5 berry weight (24.92 g) was found in Arsal on 2 July 2021 whereas the highest 5 berry weight (50.89 g) was found in Kaa El Reem (H) on 24 June 2021. Fruit diameter (mm) did not have high variations in the same location between June and July, the lowest fruit diameter values 19.67 mm were recorded in Arsal on 2 July 2021 while the highest fruit diameter values 26.33 mm were recorded in Kaa El Reem (H) on 21 June 2021. Dry matter (%) and sugar content tended to slightly increase with the season and their values almost followed a similar trend. The lowest dry matter (15.25%) was found in Kaa El Reem (H) on 21 June whereas the highest dry matter (19.54%) was found in Arsal on 2 July 2021. The lowest TSS (14.45 °Brix) was found in Arsal on 2 July 2021 whereas the highest TSS (18.7 °Brix) was found in Kaa El Reem (H) on 28 June 2021. Only titratable acidity (g/L) tended to decrease with harvest date, the lowest TA (6.75 g/L) was found in Arsal on 8 July 2021 whereas the highest TA (9.6 g/L) was found in Kaa El Reem (H) on 21 June 2021.

**Figure 2:**
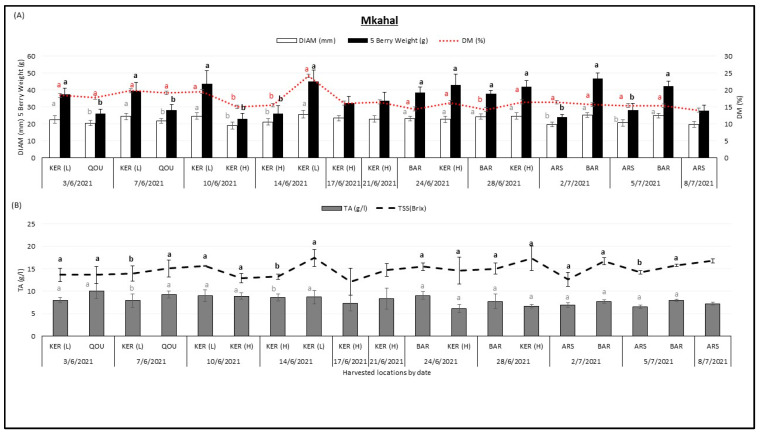
Variation of fruit diameter DIAM (mm), 5 berry weight (g), dry matter DM (%) (**A**), titratable acidity TA (g/L), and total soluble solids TSS (°Brix) (**B**), in the “Mkahal” cherry variety between different locations Qoussaya (QOU), Kaa El Reem high altitude (KER H), Kaa El Reem low altitude (KER L), Barqa (BAR), and Arsal (ARS) over the harvesting season. While comparing different locations at the same date, mean values followed by the same letter are not significantly different at *p* ≤ 0.05 level. Variables were considered significant <0.05 risk level.

**Figure 3:**
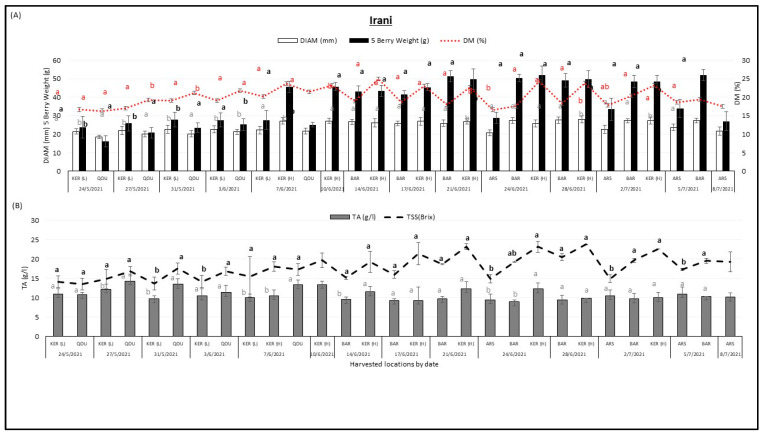
Variation of fruit diameter DIAM (mm), 5 berry weight (g), dry matter DM (%) (**A**), titratable acidity TA (g/L), and total soluble solids TSS (°Brix) (**B**), in “Irani” cherry variety between different locations Qoussaya (QOU), Kaa El Reem high altitude (KER H), Kaa El Reem low altitude (KER L), Barqa (BAR), and Arsal (ARS) over the harvesting season. While comparing different locations at the same date, mean values followed by the same letter are not significantly different at *p* ≤ 0.05 level. Variables were considered significant <0.05 risk level.

Figure 2 presents the variation of fruit diameter DIAM (mm), 5 berry weight (g), dry matter DM (%), titratable acidity TA (g/L) and total soluble solids TSS (°Brix) in “Mkahal” cherry variety between different locations over the harvesting season. «Mkahal” variety was harvested from Qoussaya, Kaa El Reem high altitude, Kaa El Reem low altitude, Barqa, and Arsal between 3 June 2021 and 8 July 2021. It was observed that the 5 berry weight (g) of the “Mkahal” variety tended to increase with the season. The lowest 5 berry weight (23.09 g) was found in Kaa El Reem (H) on 10 June 2021 whereas the highest 5 berry weight (46.73 g) was found in Barqa on 2 July 2021. Fruit diameter (mm) tended to slightly increase between June and July, the lowest fruit diameter values (19.17 mm) were recorded in Kaa El Reem (H) on 10 June 2021 while the highest fruit diameter values 25.78 mm were recorded in Kaa El Reem (L) on 14 June 2021. Dry matter (%) and sugar content had important differences between locations and their values almost followed a similar trend. The lowest dry matter (14.11%) was found in Arsal on 8 July 2021 whereas the highest dry matter (24.24%) was found in Kaa El Reem (L) on 14 June 2021. The lowest TSS (12.1 °Brix) was found in Kaa El Reem (H) on 17 June 2021 whereas the highest TSS (17.43 °Brix) was found in Kaa El Reem (L) on 14 June 2021. Only titratable acidity (g/L) tended to decrease with harvest date, the lowest TA (6.16 g/L) was found in Kaa El Reem (H) on 24 June 2021 whereas the highest TA (10 g/L) was found in Qousaya on 3 June 2021. When comparing the “Mkahal” variety in the same harvest date between two different locations, significant differences were observed as shown in Figure 3.

Figure 3 shows the variation of fruit diameter DIAM (mm), 5 berry weight (g), dry matter DM (%), titratable acidity TA (g/L), and total soluble solids (°Brix) in “Irani” cherry variety between different locations over the harvesting season. The “Irani” variety was harvested from Qoussaya, Kaa El Reem high altitude, Kaa El Reem low altitude, Barqa, and Arsal between 24 May 2021 and 8 July 2021.

It was observed that the 5 berry weight (g) of the “Irani” variety tended to increase with the season. The lowest 5 berry weight (16.07 g) was found in Qousaya on 24 May 2021 whereas the highest 5 berry weight (51.9 g) was found in Barqa on 5 July 2021. Fruit diameter (mm) tended to increase between May and July, the lowest fruit diameter values (18.53 mm) were recorded in Qousaya on 24 May 2021 while the highest fruit diameter values (28 mm) were recorded in Kaa El Reem (H) on 28 June 2021. Dry matter (%) and sugar content tended to increase with the season and their values almost followed a similar trend. The lowest dry matter (16.31%) was found in Qousaya on 24 May 2021 whereas the highest dry matter (25%) was found in Kaa El Reem (H) on 14 June 2021. The lowest TSS (13.49 °Brix) was found in Qousaya on 24 May 2021 whereas the highest TSS (23.75 °Brix) was found in Kaa El Reem (H) on 28 June 2021.

Only titratable acidity (g/L) tended to decrease with harvest date, the lowest TA (8.95 g/L) was found in Barqa on 24 June 2021 whereas the highest TA (14.28 g/L) was found in Qousaya on 27 May 2021. Significant differences were shown between locations when comparing the maturity indices of the “Irani” variety at the same harvest date. 

**Figure 4:**
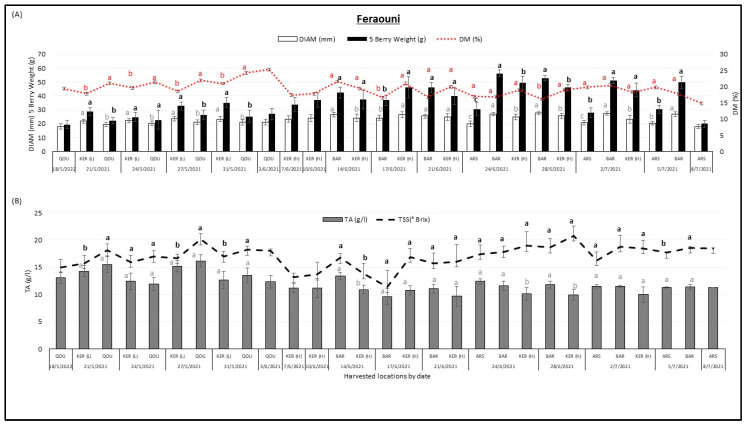
Variation of fruit diameter DIAM (mm), 5 berry weight (g), dry matter DM (%) (**A**), titratable acidity TA (g/L) and total soluble solids TSS (°Brix) (**B**), in “Feraouni” cherry variety between different locations Qoussaya (QOU), Kaa El Reem high altitude (KER H), Kaa El Reem low altitude (KER L), Barqa (BAR), and Arsal (ARS) over the harvesting season. While comparing different locations at the same date, mean values followed by the same letter are not significantly different at *p* ≤ 0.05 level. Variables were considered significant <0.05 risk level.

Figure 4 describes the variation of fruit diameter DIAM (mm), 5 berry weight (g), dry matter DM (%), titratable acidity TA (g/L), and total soluble solids (°Brix) in “Feraouni” cherry varieties between different locations over the harvesting season. The “Feraouni” variety was harvested from Qoussaya, Kaa El Reem high altitude, Kaa El Reem low altitude, Barqa, and Arsal between 18 May 2021 and 8 July 2021.

It was observed that the 5 berry weight (g) of “Feraouni” variety tended to increase with the season. The lowest 5 berry weight (19.5 g) was found in Qousaya on 8 May 2021 whereas the highest 5 berry weight (56.24 g) was found in Barqa on 24 June 2021. Fruit diameter (mm) tended to increase between May and July, the lowest fruit diameter values (17.96 mm) were recorded in Arsal on 8 July 2021 while the highest fruit diameter values (27.63 mm) were recorded in Barqa on 28 June 2021. Dry matter (%) and sugar content tended to increase with the season and their values almost followed a similar trend. The lowest dry matter (14.9%) was found in Arsal on 8 July 2021 whereas the highest dry matter (25.33%) was found in Qousaya on 3 June 2021. The lowest TSS (11.38 °Brix) was found in Barqa on 17 June 2021 whereas the highest TSS (20.79 °Brix) was found in Kaa El Reem (H) on 28 June 2021. Only titratable acidity (g/L) tended to decrease with harvest date, the lowest TA (9.63 g/L) was found in Barqa on 17 June 2021 whereas the highest TA (16.17 g/L) was found in Qousaya on 27 May 2021.

**Figure 5:**
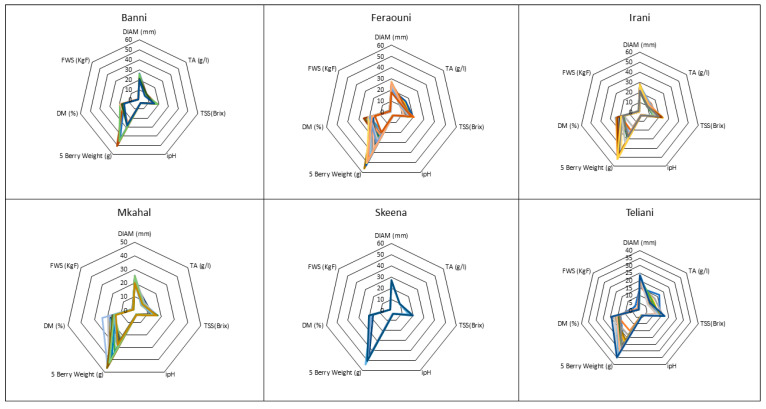
Radar showing the minimum and maximum values of maturity indices fruit diameter DIAM (mm), 5 berry weight (g), dry matter DM (%), titratable acidity TA (g/L), and total soluble solids TSS (°Brix) in cherry varieties in different locations over the harvesting season.

According to the Radar analysis presented in Figure 5, titratable acidity values for all the studied cherry varieties in all locations ranged between 6.16 and 16.5 g/L. The highest average TA value was found in “Feraouni” (12 g/L) and the lowest average TA value was found in “Mkahal” cherries (8 g/L). Regarding locations, Qousaya had the highest average TA (12.7 g/L) and Arsal had the lowest average TA (9.47 g/L).

Total soluble solids values for all the studied cherry varieties in all locations ranged between 11.21 and 23.75 °Brix. The highest average TSS value was found in “Skeena” (18.39 °Brix) and the lowest average TSS value was found in “Teliani” cherries (13.19 °Brix). As for locations, Kaa El Reem (H) had the highest average TSS (17.23 °Brix) and Kaa El Reem (L) had the lowest average TSS (14.87 °Brix). Initial pH values for all the studied cherry varieties in all locations ranged between 2.92 and 3.64. The highest average ipH value was found in “Teliani” (3.41) and the lowest average ipH value was found in “Feraouni” cherries (3.17). As for locations, Arsal had the highest average ipH (3.35) and Qousaya had the lowest average ipH (3.12).

The 5 Berry Weight values for all the studied cherry varieties in all locations ranged between 15.14 and 56.24 g. The highest average 5 berry weight value was found in “Skeena” (49.86 g) and the lowest average value was found in “Teliani” cherries (26.68 g). As for locations, Barqa had the highest average (44.32 g) and Qousaya had the lowest average (22.88 g).

Fruit diameter values for all the studied cherry varieties in all locations ranged between 15.27 and 28 mm. The highest average diameter value was found in “Skeena” (26.73 mm) and the lowest average diameter value was found in “Teliani” cherries (21.15 mm). As for locations, Barqa had the highest average diameter (25.78 mm) and Qousaya had the lowest average diameter (19.68 mm). Dry matter values for all the studied cherry varieties in all locations ranged between 13.18 and 25.33%. The highest average DM value was found in “Irani” (20.09%) and the lowest average DM value was found in “Teliani” cherries (15.94%). As for locations, Qousaya had the highest average DM and Arsal and Barqa had the lowest average DM (17.33%).

Firmness with skin values for all the studied cherry varieties in all locations ranged between 0.53 and 3.83 KgF. The highest average FWS value was found in “Feraouni” (1.38 KgF) and the lowest average FWS value was found in ”Banni” cherries (1.1 KgF). As for locations, Qousaya had the highest average FWS (1.81 KgF) and Kaa El Reem (H) had the lowest average FWS (1.09 KgF). The firmness with skin (FWS) measurements fell between 0.53 and 3.83 KgF. The “Feraouni” cherries had the highest average FWS value at 1.38 KgF, while the “Banni” cherries had the lowest average FWS value at 1.1 KgF. Among the locations, Qousaya had the highest average FWS at 1.81 KgF, whereas Kaa El Reem (H) had the lowest average FWS at 1.09 KgF.

Analysis of fruit firmness revealed a high variation of values inside the same variety, as per Table 2 but overall, “Feraouni” cherries had the highest fruit firmness average (1.38 KgF) over the harvesting seasonm followed by “Mkahal” (1.31 KgF), “Irani” (1.2 KgF), “Teliani” (1.18 KgF), “Skeena” (1.19 KgF), and “Banni” (1.1 KgF). Analysis of initial pH revealed a slight variation of values inside the same variety for “Mkahal”, “Banni”, and “Skeena” and a high variation of values for “Teliani”, “Feraouni”, and “Irani”. The lowest initial pH average (3) was recorded in “Feraouni” and the highest initial pH average (3.64) was recorded in “Teliani”.

**Figure 6:**
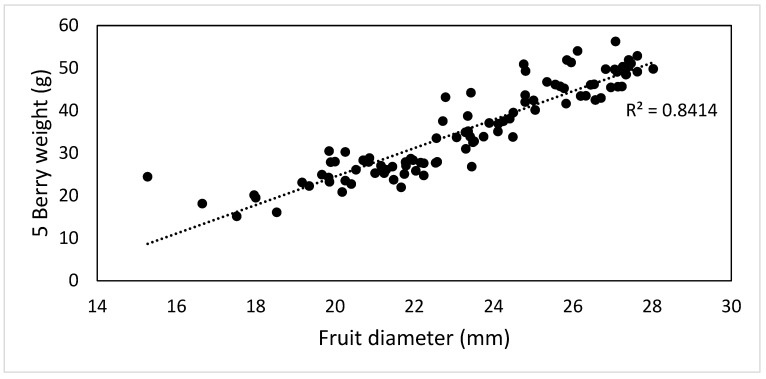
Correlation between 5 berry weight (g) and fruit diameter (mm).

A high positive correlation (R^2^ = 0.84) was observed only between the 5 berry weight (g) and the fruit diameter (mm) (Figure 6).

**Figure 7:**
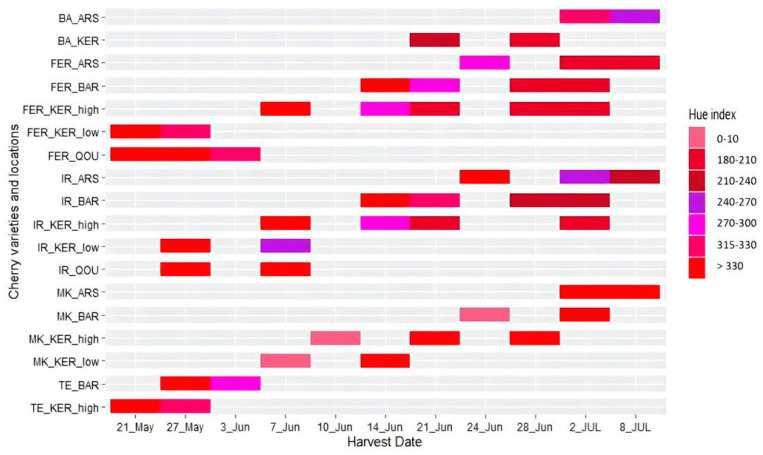
Heatmap illustrating color development of the cherry varieties Banni (BA), Feraouni (FER), Irani (IR), Mkahal (MK), and Teliani (TE) in their growing locations Arsal (ARS), Kaa El Reem low and high altitudes (KER), Qoussaya (QOU), and Barqa (BAR) during the ripening season.

Based on Figure 7, a high variability in cherry skin color was observed when comparing different varieties or the same variety in different locations. The effect of altitude on skin color was observed when comparing the same variety harvested from different locations at the same date. On the 21st of May, “Feraouni” harvested from Kaa El Reem (low) and Qousaya had close hue index values, which was not the case when the same cherries were harvested a week later, on 27 May 2021. On the other hand, different hue index values were observed for “Feraouni” harvested from Barqa and Kaa El Reem (high) on 14 June 2021; however, on 28 June 2021, cherries harvested from the same locations had close hue index values. The above heat map shows the different potential hue index values that can be reached according to each variety at the end of the harvest season. On 8 July 2021, “Mkahal” variety had hue index values comparable to “Feraouni”, “Teliani”, or “Irani” at the early harvest stages.

### 2.2. Phytochemical Analysis

“Banni”, “Feraouni”, “Irani”, and “Mkahal” cherry varieties were tested for phytochemical composition (Table 3). Total phenolic content (GAE) ranged between 458 and 719 (µg/mL) for all varieties in all locations. DPPH % in cherry varieties ranged between 62.35% and 85.34%. TEAC content was between 49.45 and 666.71 whereas FRAP content was between 1008.67 (µM) and 1675.42 (µM). “Banni” cherries harvested from Arsal had significantly higher content compared to those harvested in Kaa El Reem in Gallic acid and FRAP but there was no significant difference in DPPH inhibition or TEAC content. “Feraouni” cherries harvested from Kaa El Reem had significantly higher concentrations compared to those harvested in Barqa in Gallic acid and FRAP but there was no significant difference in DPPH inhibition or TEAC concentrations. “Irani” cherries harvested from Barqa and Kaa El Reem did not have significant differences in total phenolic content (GAE), DPPH, TEAC, and FRAP when comparing the two locations.

Analysis of total phenolic content (GAE), DPPH, TEAC and FRAP the evaluated cherry varieties regardless their harvested locations revealed that there was no significant difference for total phenolic content (GAE) in “Banni”, “Feraouni”, “Irani”, and “Mkahal” varieties (Table 4). DPPH inhibition% and TEAC content were the highest in “Mkahal” variety (85.34 DPPH inhibition% and 66.71 µM TAEC) and the lowest content was found in “Banni” variety (72.07 DPPH inhibition% and 56.75 µM TAEC). No significant difference was observed when comparing “Feraouni” (83.11 DPPH inhibition% and 65.04 µM TAEC) and “Irani” (78.63 DPPH inhibition% and 62.05 µM TAEC). Concerning FRAP content, “Irani” variety had the highest levels (1660.99 µM) and it was not significantly different when compared to “Feraouni” (1552.45 µM) and “Banni” (1369.52 µM). Only the “Mkahal” variety had a significant lower FRAP content (1209.67 µM). The “Feraouni” and “Irani” varieties exhibited considerably greater levels of total anthocyanins compared to the “Mkahal” and “Banni” cultivars, which displayed the lowest total anthocyanin levels.

## 3. Discussion

Sweet cherry growers rely on important characteristics that offer valuable information regarding fruit maturity such as total soluble solids, titratable acidity, and fruit weight [17].

According to Pérez-Sánchez et al. [17], fruit firmness values of Spanish sweet cherry varieties ranged from 7.66 to 15.28 N, “Blanca de Provenza,” “Corazón Serrano,” “Burlat”, and “Monzón” being the cultivars with the firmest fruits. This quality parameter is important for the determination of susceptibility of cherry fruits to mechanical damage, picking, and transportation. In this study, the “Feraouni” variety had the higher average of firmness with skin values (1.38 KgF) followed by “Mkahal” (1.31 KgF), “Irani” (1.2 KgF), “Skeena” (1.19 KgF), “Banni” (1.1 KgF), and “Teliani” (0.96 KgF). It was reported that late varieties had firmer fruits compared to early varieties [18] and this was the case of “Teliani” variety, which is considered an early harvested cultivar in Lebanon compared to the “Feraouni” cultivar, which is harvested relatively late. Pérez-Sánchez et al. [17] remarked that irrespective to the cultivars of sweet cherries, there were reductions in fruit firmness during ripening and storage time as a consequence of climatic factors, for instance, temperature and precipitation. Similar results were observed in this study and were reflected in Table 2 by the large difference between minimum and maximum values of fruit firmness over the harvesting period.

With respect to the total soluble solid ratio for sweet cherries, for commercial purposes, minimum levels were set. Palou [19] realized that TSS levels in sweet cherries must exceed 14.0° Brix for the marketing of high-quality sweet cherries [19]. Vursavus et al. [20] and Usenik et al. [18] analyzed soluble solids in sweet cherries. TSS levels recorded in their study varied between 14.4 and 13.84 °Brix for “Van” variety, whereas other varieties such as “Ambrunés” recorded higher TSS levels around 17 °Brix [21]. In this study, the Lebanese varieties showed significant differences in their average TSS levels over the harvested season. “Teliani” (13.19 °Brix) had the lower levels followed by “Mkahal” (14.78 °Brix), “Banni” (16.29 °Brix), “Feraouni” (16.94 °Brix), “Irani” (17.91 °Brix), and “Skeena” (18.39 °Brix).

The tested Lebanese cherry cultivars also differed in their titration acidities (TA). “Mkahal” (8 g/L) had the lower levels followed by “Banni” (8.7 g/L), “Teliani” (9.05 g/L), “Skeena” (9.5 g/L), “Irani” (10.8 g/L), and “Feraouni” (12 g/L). According to Szpadzik et al. [22], malic acid is the most prevalent organic acid in various cherry cultivars. In sweet cherries, the soluble solid content (SSC) and titration acidity (TA) are both very important traits since the taste and consumer’s choice is mostly related to an equilibrium between the sugar and the acid contents. These traits are influenced by the weather conditions during the ripening phase of cherry fruit. Sugar synthesis increases with sunlight and high temperatures [22].

As for dry matter, “Teliani” (15.94%) had the lower levels followed by “Mkahal” (16.92%), “Banni” (17.28%), “Skeena” (18.7%), “Feraouni” (19.32%) and “Irani” (20.09%). As for fruit diameter, “Teliani” (21.15 mm) had the lower levels followed by “Mkahal” (22.8 mm), “Banni” (22.88 mm), “Feraouni” (23.22 mm), “Irani” (24.33 mm) and “Skeena” (26.73 mm). As for 5 berry weight, “Teliani” (26.68 g) had the lower levels followed by “Mkahal” (35.11 g), “Feraouni” (36 g), “Banni” (36.81 g), “Irani” (37.33 g), and “Skeena” (49.86 g). The weight and size of cherry fruits are the main characteristics that control the commercial value of this fruit. Consumers state that fruits of a larger size and darker color are more appealing [22]. Researchers have investigated the complex sweet cherry genome, an important step to identify candidate genes and metabolic pathways with major roles in the determination of fruit quality characteristics [23]. Quantitative trait loci have been identified for many maturity indices such as fruit weight, firmness, fruit size, skin and flesh color, and fruit cracking tolerance [24]. Other factors such as rootstocks, growth regulators application, agricultural practices, and orchard management have an important impact on productivity. Choosing the right rootstock × scion combination is widely studied due to its importance for productivity [25] and its effects on fruit quality [26]. For instance, Goncalves et al. (2006) studied the scion–rootstock interaction and how it can affect the physiology and fruit quality of sweet cherry [27]. They demonstrated that the rootstock genotype influences water relations and photosynthesis; moreover, fruit quality regulation was mainly dependant on cultivar genotype with a significant effect of rootstocks. According to Vangdal et al. [28], an application of 0.5% of Ca Cl2 between petal fall and until 2 weeks before harvest increased SSC and phenolics and reduced fruit decay and cuticular cracks. Studies showed that an application of giberrelic acid (GA3) at the green to straw yellow stage had an impact on fruits that were softer but no impact on SSC [29].

Regarding the phytochemical composition, cherries are known to possess high levels of antioxidants, which are beneficial for human health. Gallic acid, DPPH (2,2-diphenyl-1-picrylhydrazyl), TEAC (Trolox Equivalent Antioxidant Capacity), FRAP (Ferric Reducing Antioxidant Power), and total anthocyanins are some of the commonly used parameters to measure antioxidant activity in fruits. El Baji et al. [30] carried out research on four cherry cultivars that were grown in various agro-ecological conditions in Morocco. The study revealed that the production of anthocyanins may be affected by the geographical location, especially in areas with higher precipitation. However, the agro-ecological conditions did not have any impact on the total polyphenol, flavonoid, and antioxidant activity of the sweet cherries produced in the two locations that were studied. The levels of gallic acid, DPPH (2,2-diphenyl-1-picrylhydrazyl) activity, TEAC (Trolox Equivalent Antioxidant Capacity), FRAP (Ferric Reducing Antioxidant Power), and total anthocyanins found in sweet cherry cultivars can be influenced by the location where they are grown. Various factors such as climate, soil type, altitude, and light intensity can affect the chemical composition of cherries, which in turn affects their antioxidant and nutrient content.

For instance, a study by Slatnar et al. [31] investigated the antioxidant capacity and phenolic composition of five sweet cherry cultivars grown in three different locations in Slovenia. The study found that the total phenolic content and antioxidant capacity of cherries varied significantly among the different locations. The cherries grown in the coastal region had the highest levels of gallic acid, DPPH activity, TEAC, and FRAP, while those grown in the continental region had the highest total anthocyanin content.

Another study by Zhang et al. [32] analyzed the phenolic compounds and antioxidant activity of sweet cherries from three different regions in China. The results showed that the levels of gallic acid, DPPH, and total anthocyanins were significantly higher in cherries grown in the Xinjiang region compared to those grown in the Shanxi and Shandong regions. The authors attributed this difference to variations in climate and soil conditions among the regions.

The total phenolic compounds and antioxidant activity can be affected by various factors, including the dehydration process. In their studies conducted in 2022 and 2023, Özcan et al. [33,34] discovered that the untreated (control) samples of Hawthorn (*Crataegus* spp.) and sandal strawberry tree fruit had higher levels of total phenolic compounds and antioxidant activity than the dehydrated ones. They also found a linear correlation between the total phenolic content and antioxidant values of the samples.

## 4. Materials and Methods

Handpicked cherry berries from commercial Lebanese orchards situated in different locations including Arsal (2080 m), Qousaya (1130 m), Kaa El Reem (1280 m and 1600 m), and Barqa (1930 and 1980 m), as outlined in Table 5, were analyzed. The cherries, which comprised traditional varieties such as “Feraouni”, Banni, “Irani”, “Mkahal”, “Teliani”, and the recently introduced “Skeena” variety, were harvested twice a week between May 2021 and July 2021. In each studied orchard, trees representing 10–15% were selected, and 30 fruits were handpicked from each tree early in the morning, coded based on the location, farm name, row number, and tree number, and placed in paper bags. These cherry samples were then taken to the laboratory on the same day for postharvest physiochemical analysis. Cherry samples were visually inspected at the laboratory to ensure that they were not subjected to any damage during transportation. If any of the cherries were damaged, they were removed from the samples.

### 4.1. Experimental Study

Several measurements were conducted on cherry fruits and various fruit parameters were assessed. The measurements included:

- Recording the weight of every 5 fruits separately, for 6 repetitions of 5 fruits per tree.
- Conducting colorimetric measurements of 30 fruits per tree using the AgroColor colorimeter (Agrosta, Serqueux, France).
- Measuring the diameter of 30 fruits per tree using a caliper.
- Determining the firmness of 30 fruits per tree using a QA FT 327 Fruit Pressure Tester (QA supplies, Norfolk, VA, USA), equipped with a 3-mm tip, and expressing the values in (KgF).
- Dry matter content of the cherry fruits, kernels from 12 fruits were removed, and 6 repetitions of 2 fruits per tree were weighed before and after being dried in an oven at 65 °C for 36 h, until reaching a constant weight. The results were expressed as a percentage of the original weight and recorded as an average of six individual values per tree.
- To measure the total soluble solids (TSS) of the cherry fruits, the remaining 18 fruits were blended into juice and tested for TSS expressed as a degree Brix by placing one or two drops of the juice on the prism of a digital Atago PAL-1 refractometer.
- The initial pH of the juice was also recorded using a HANNA HI2211 pH meter.
- Titratable acidity: 5 mL of the juice was diluted with 95 mL of dH2O to measure the pH using a HANNA HI2211 pH meter. The titratable acidity was conducted using 0.1 M NaOH until reaching a pH of 8.1. Titratable acidity was calculated as the number of milliliters of NaOH used multiplied by an appropriate factor using this equation (X*75*0.1)/5)*100, where X is the amount of NaOH used, 75 is the tartaric acid conversion factor.

### 4.2. Phytochemical Assays

- Total phenolic content: Total phenolic content was determined with a Folin Ciocâlteu Phenolic Content Quantification Assay Kit (BioQuoChem, Spain) in a 20% (*w*/*v*) cherry juice solution in a 96-well microplate according to the manufacturer’s instructions. Each sample and standard was tested in triplicate and repeated three times. Gallic (GAE) acid was used as the reference standard and results were expressed as µg of gallic acid equivalent per ml of solution (GAE µg/mL). Absorbance was read at 700 nm at 37 °C.
- Ferric Reducing Antioxidant Power Assay: The ferric ion reducing antioxidant power (FRAP) was determined using a standardized capacity kit (BioQuoChem, Asturias, Spain). The reduction of the ferric complex by the extracts at an acidic pH was quantified. The diluted sample (10 µL) was mixed with 220 µL of FRAP working solution and left to react for 4 min. The absorbance was then measured using a microplate reader at 593 nm. The antioxidant activity was expressed as µM of iron(II) equivalent (µM iron (II)).
- DPPH Radical Scavenging Assay: DPPH (2,2-Diphenyl-1-picrylhydrazyl) is a stable free radical that can be used to measure the radical scavenging activity of antioxidants. Measurements of DPPH radical scavenging activity were performed using a commercial kit, following the manufacturer’s instructions. Briefly, samples were appropriately diluted in DMSO and mixed with the DPPH solution provided by the kit. The total antioxidant capacity (TAC) was determined by measuring absorbance at 517 nm through a multimode microplate reader (Thermofihser Scientific, Waltham, MA, USA, Varioskan LUX), and calculating the corresponding percentage of the inhibition of the radical DPPH as reported in the kit instructions. The DPPH radical scavenging activity of sample was expressed as Trolox equivalent antioxidant capacity (TEAC, μM).
- Anthocyanins: Total anthocyanin content was measured using the BQC kit according to the manufacturer’s recommendations (BioQuoChem, Asturias, Spain). A measure of 20 μL of cherry extracts (10 mg/mL) was mixed with 220 μL of Reagent A or 220 μL of Reagent B in 96-well clear bottom plates. Then, the plates were shaken at 200 rpm, left for 10 min, and the absorbance measured at 510 and 700 nm using a multimode microplate reader (Thermofihser Scientific, USA, Varioskan LUX). AC values expressed as the means of 3 replicate measurements.

### 4.3. Statistical Analysis

Statistical analysis was undertaken when samples were obtained from various sites on the same date for the same variety. After the data were entered and cleaned from extreme values, statistical comparison of all parameters was performed using *t*-tests and one-way ANOVA tests. Differences were considered statistically significant for *p*-values < 0.05 using RStudio statistical software. Radar analysis was performed over all harvesting dates to observe the clustering behavior of the varieties and correlations between maturity indices. Shapiro–Wilk normality tests and studentized residual plots were used to test error assumptions of variance analysis, including random, homogenous, and normal distribution of error. Statistical comparison of maturity indices under different locations by variety was performed using one-way ANOVA parametric test followed by Student–Newman–Keuls (SNK) post hoc test. As a non-parametric alternative to ANOVA, the Kruskal–Wallis test was used followed by Dunn’s test. Differences between locations for the same variety were considered statistically significant for *p*-values < 0.05 using R Studio statistical software.

## 5. Conclusions

In conclusion, the location where sweet cherries are grown can have a significant impact on their antioxidant capacity and phenolic composition. Therefore, it is important to consider the origin of sweet cherries when evaluating their health benefits and nutritional value. Further research should aim to study cultivar-specific strategies that can improve fruit quality and the level of health-related compounds. The interaction of genetics, agricultural practices, rootstocks, environmental factors, growth regulators, storage techniques, and new preservation technologies has a great impact on final fruit quality and, thus, needs to be optimized. 

## Figures and Tables

**Table 1:** Variation of fruit diameter DIAM (mm), 5 berry weight (g), dry matter DM (%), titratable acidity TA (g/L), and total soluble solids (°Brix) in Banni and Skeena cherry varieties between different locations Kaa El Reem high altitude (KER H) and Arsal (ARS) over the harvesting season. While comparing the two varieties in different locations at the same date, mean values followed by the same letter are not significantly different at *p* ≤ 0.05 level. Variables were considered significant <0.05 risk level.

Harvest Date	Variety	Location	DIAM (mm)	TA (g/L)	TSS (°Brix)	5 Berry Weight (g)	DM (%)
21 June 2021	Banni	KER (H)	26.33 ± 1.19 a	9.6 ± 0.28 a	16.6 ± 1.27 a	43.46 ± 3.17 a	15.25 ± 1.06 a
21 June 2021	Skeena	KER (H)	26.45 ± 1.15 a	9.57 ± 0.59 a	17.17 ± 0.33 a	46.06 ± 2.41 a	17 ± 1.41 a
24 June 2021	Banni	KER (H)	24.76 ± 1.54 b	9.05 ± 0.08 a	17.86 ± 0.2 a	50.89 ± 3.49 a	16.54 ± 3.2 a
24 June 2021	Skeena	KER (H)	26.12 ± 1.8 a	9.27 ± 0.84 a	16.53 ± 1.1 a	54.01 ± 3.03 a	18.43 ± 1.2 a
28 June 2021	Banni	KER (H)	25.68 ± 1.19 b	8.1 ± 0.21 a	18.7 ± 0.14 a	45.69 ± 3.28 a	19.41 ± 2.14 a
28 June 2021	Skeena	KER (H)	27.12 ± 1.1 a	9.67 ± 0.49 a	20.05 ± 0.36 a	49.08 ± 1.8 a	18.89 ± 1.4 a
2 July 2021	Banni	ARS	19.67 ± 1.99 b	9.5 ± 0.14 a	14.45 ± 2.05 b	24.92 ± 2.64 b	19.54 ± 2.6 a
2 July 2021	Skeena	KER (H)	27.26 ± 1.04 a	9.5 ± 1.41 a	19.83 ± 0.4 a	50.31 ± 2.64 a	20.47 ± 1.04 a
5 July 2021	Banni	ARS	20.85 ± 2.11	9.3 ± 0.53	14.9 ± 0.1	27.92 ± 3.79	17.63 ± 3.06
8 July 2021	Banni	ARS	20.00 ± 1.13	6.75 ± 0.01	15.25 ± 0.07	27.98 ± 2.22	15.36 ± 0.87

**Table 2:** Average cherry fruit firmness and ipH values of the evaluated varieties over the harvesting season. Mean values followed by the same letter are not significantly different at *p* ≤ 0.05 level. Variables were considered significant <0.05 risk level.

Variety	Location	ipH	FWS (KgF)
Banni	Kaa El Reem (H)	3.4 bc ± 0.12	0.99 bc ± 0.06
Banni	Arsal	3.4 b ± 0.1	1.22 bc ± 0.02
Feraouni	Qousaya	3 g ± 0.08	1.72 b ± 0.37
Feraouni	Kaa El Reem (L)	3.1 efg ± 0.06	1.39 bc ± 0.22
Feraouni	Kaa El Reem (H)	3.3 bcde ± 0.1	1.13 bc ± 0.32
Feraouni	Barqa	3.2 def ± 0.06	1.44 bc ± 0.2
Feraouni	Arsal	3.2 cdef ± 0.09	1.3 bc ± 0.23
Irani	Qousaya	3.1 fg ± 0.09	1.65 b ± 0.45
Irani	Kaa El Reem (L)	3.3 bcd ± 0.05	1.11 bc ± 0.15
Irani	Kaa El Reem (H)	3.3 bcde ± 0.08	1.12 bc ± 0.17
Irani	Barqa	3.3 bcd ± 0.09	1.05 bc ± 0.34
Irani	Arsal	3.3 bcd ± 0.08	1.21 bc ± 0.21
Mkahal	Qousaya	3.3 bcd ± 0.02	1.69 bc ± 0.26
Mkahal	Kaa El Reem (L)	3.3 bcd ± 0.11	1.43 bc ± 0.09
Mkahal	Kaa El Reem (H)	3.4 bcd ± 0.08	1.21 bc ± 0.29
Mkahal	Barqa	3.3 bcd ± 0.07	1.25 bc ± 0.2
Mkahal	Arsal	3.4 b ± 0.07	1.24 bc ± 0.07
Teliani	Qousaya	3.3 bcd ± 0.05	2.35 a ± 1.33
Teliani	Kaa El Reem (L)	3.4 bc ± 0.12	0.78 c ± 0.15
Teliani	Kaa El Reem (H)	3.4 b ± 0.06	0.72 c ± 0.07
Teliani	Barqa	3.4 b ± 0.06	0.92 bc ± 0.15
Teliani	Arsal	3.6 a ± 0.01	1.07 bc ± 0.01
Skeena	Kaa El Reem (H)	3.2 cdef ± 0.09	1.19 bc ± 0.05

**Table 3:** Total phenolic content (GAE), DPPH, TEAC, and FRAP in “Banni”, “Feraouni”, “Irani”, and “Mkahal” varieties according to their locations. Mean values followed by the same letter are not significantly different at *p* ≤ 0.05 level. Variables were considered significant <0.05 risk level.

Variety	Location	GAE (µg/mL)	DPPH Inhibition %	TEAC (µM)	FRAP (µM)
Banni	Kaa El Reem	458 ± 23.4 b	62.35 ± 11.21	49.45 ± 8.41	1075.96 ± 24.04 b
	Arsal	581 ± 5.9 a	81.79 ± 0.53	64.05 ± 0.40	1663.08 ± 24.92 a
Feraouni	Barqa	517 ± 43.6 b	85.27 ± 0.067	66.66 ± 0.05	1008.67 ± 12.67 b
	Kaa El Reem	719.27 ± 15.26 a	80.95 ± 1.26	63.42 ± 0.95	2036.23 ± 111.53 a
Irani	Barqa	650 ± 22.8	82.06 ± 0.46	64.25 ± 0.35	1675.42 ± 86.00
	Kaa El Reem	627.55 ± 13.84	75.2 ± 1.97	59.85 ± 1.39	1646.55 ± 35.80
Mkahal	Kaa El Reem	520.68 ± 37.39	85.34 ± 0.15	66.71 ± 0.11	1209.67 ± 124.24

**Table 4:** The levels of total phenolic content (GAE), DPPH, TEAC, FRAP, and total anthocyanins found in the “Banni”, “Feraouni”, “Irani”, and “Mkahal” cultivars during the harvest season irrespective of their origin. Mean values followed by the same letter are not significantly different at *p* ≤ 0.05 level. Variables were considered significant <0.05 risk level.

Variety	GAE (µg/mL)	DPPH Inhibition%	TEAC (µM)	FRAP (µM)	Total Anthocyanins (mg/L)
Banni	519.5 ± 29.89	72.07 ± 6.64 c	56.75 ± 4.98 c	1369.52 ± 138.87 ab	121.21 ± 11.89 c
Feraouni	618.14 ± 27.42	83.11 ± 1.1 ab	65.04 ± 0.83 ab	1522.45 ± 144.65 a	593.764 ± 21.63 a
Irani	638.78 ± 11.52	78.63 ± 1.6 bc	62.05 ± 1.2 bc	1660.99 ± 30.28 a	367.870 ± 15.53 b
Mkahal	520.68 ± 37.39	85.34 ± 0.15 a	66.71 ± 0.11 a	1209.67 ± 124.24 b	55.221 ± 5.43 c

**Table 5:** Variety names and places, GPS coordinates, elevations, and collection sample dates.

Variety	Location	Longitude	Latitude	Altitude (m)	Harvest Date
Feraouni	Qousaya	33.807742	36.02482	1130	May–June
	Kaa El Reem (Low)	33.888591	35.872562	1280	May
	Kaa El Reem (High)	33.888591	35.872562	1600	June–July
	Barqa	34.215077	36.132286	1930	June–July
	Arsal	34.095397	36.409924	2080	June–July
Banni	Kaa El Reem (High)	33.888591	35.872562	1600	June
	Arsal	34.095397	36.409924	2080	July
Irani	Qousaya	33.807742	36.02482	1130	May–June
	Kaa El Reem (Low)	33.888591	35.872562	1280	May–June
	Kaa El Reem (High)	33.888591	35.872562	1600	June–July
	Barqa	34.215077	36.132286	1930	June–July
	Arsal	34.095397	36.409924	2080	June–July
Mkahal	Qousaya	33.807742	36.02482	1130	June
	Kaa El Reem (Low)	33.888591	35.872562	1280	June
	Kaa El Reem (High)	33.888591	35.872562	1600	June
	Barqa	34.215077	36.132286	1930	June–July
	Arsal	34.095397	36.409924	2080	July
Teliani	Kaa El Reem (Low)	33.888591	35.872562	1280	May
	Kaa El Reem (High)	33.888591	35.872562	1600	May
	Barqa	34.215077	36.132286	1930	May–June
	Arsal	34.095397	36.409924	2080	June
Skeena	Kaa El Reem (High)	33.888591	35.872562	1600	June–July

## Data Availability

All data included in the main text.
